# The implications of trade liberalization for diet and health: a case study from Central America

**DOI:** 10.1186/1744-8603-5-5

**Published:** 2009-07-28

**Authors:** Anne Marie Thow, Corinna Hawkes

**Affiliations:** 1 Menzies Centre for Health Policy, Victor Coppleson Bldg (D02), University of Sydney, Sydney, NSW 2006, Australia; 2 Research Fellow, International Food Policy Research Institute, current affiliation: Research Fellow, School of Public Health, University of Sao Paolo, Sao Paulo, Brazil

## Abstract

**Background:**

Central America has undergone extensive trade liberalization over the past two decades, and has recently signed a Free Trade Agreement with the United States. The region is also experiencing a dual burden of malnutrition with the growth of dietary patterns associated with the global 'nutrition transition'. This study describes the relationship between trade liberalization policies and food imports and availability, and draws implications for diet and health, using Central America as a case study region.

**Methods:**

Changes in tariff and non-tariff barriers for each country were documented, and compared with time-series graphs of import, production and availability data to show the outcome of changes in trade policy in relation to food imports and food availability.

**Results:**

Changes in trade policy in Central America have directly affected food imports and availability via three avenues. First, the lowering of trade barriers has promoted availability by facilitating higher imports of a wide range of foods. Second, trade liberalization has affected food availability through promoting domestic meat production. Third, reductions in barriers to investment appear to be critical in expansion of processed food markets. This suggests that changes in trade policies have facilitated rising availability and consumption of meat, dairy products, processed foods and temperate (imported fruits) in Central America.

**Conclusion:**

This study indicates that the policies of trade liberalization in Central American countries over the past two decades, particularly in relation to the United States, have implications for health in the region. Specifically, they have been a factor in facilitating the "nutrition transition", which is associated with rising rates of obesity and chronic diseases such as cardiovascular disease and cancer. Given the significant cost of chronic disease for the health care system, individuals and the wider community, it is critical that preventive health measures address such upstream determinants of poor nutrition.

## Background

In what has been termed the 'nutrition transition', the developing world is currently experiencing rapid shifts in food availability and consumption. Diets based on local staples are giving way to rising consumption of fats, animal products and sweeteners, at the same time as physical activity levels are declining. The consequences of this dietary and lifestyle change are being seen throughout the developing world in rapidly increasing rates of obesity, diabetes and other non-communicable disease [1,2]. The causes of this dietary transition are diverse, and while individual lifestyle choices play a role, macro factors are critical in shaping the food environment through their influence on food availability and price [3].

A core upstream driver of the nutrition transition is globalization [4-6]. Reductions in barriers to trade, the growth of transnational food companies, foreign direct investment and liberalization of media advertising have all been highlighted as inter-related avenues through which globalization is driving the nutrition transition [7]. In particular, reductions in barrier to trade -trade liberalization – is one of the processes of globalization commonly cited as contributing to dietary change [8]. In the literature on globalization, diet, obesity and chronic diseases, it is often assumed that trade liberalization encourages greater imports of "western" foods, thus changing food consumption patterns and, therefore, diets and health. However, there are few studies that actually attempt to identify if there is a direct link between trade policy and the food environment from a public health perspective [9].

This paper investigates the impact of trade policy change on food imports and availability in Central America, in the context of regional changes in diet and health and a progressive liberalization agenda.

The region provides a particularly useful case study for several reasons. First, since the early 1990s, Central America has undergone a period of trade liberalization with its leading trading partner, the United States, for a range of foodstuffs. This liberalization entered a new phase in 2004 with the signing of the US-Central American Free Trade Agreement (CAFTA), which has been implemented progressively – albeit following different timeframes in different countries – since 2005 [10,15]. Second, health and dietary data from the region suggest that Central American countries are at varying stages of the nutrition transition and are experiencing rising rates of diet-related chronic disease, with associated health and economic implications [11-13]. Third, information and data are available about trade policies and trade between Central America and the United States. And fourth, the countries have similarities and differences that provide the opportunity for some cross-country analysis.

This article focuses on one key aspect of trade liberalization, the reduction of barriers to food imports, with particular reference to the impacts of trade policy on food imports from the regions' key trading partner, the United States.

## Methods

### Data sources

Food availability and production data were sourced from the FAOSTAT database [14,15]. FAO food balance sheet and supply utilization account data were used to provide information on food availability (for human consumption), and the ProdSTAT database provided information on domestic production. While FAO data have some limitations associated with necessary estimates made to compensate for limited data, it is generally accepted that they provide a useful indication of the food supply – particularly in relation to trends over time (see [16] for further detail).

Food export data from the United States Department of Agriculture Foreign Agricultural Service [17] were extracted for US exports into Central American countries at the internationally consistent "6-digit" level of the Harmonized Tariff Schedule, which can be matched exactly with information on changes in tariff and non-tariff barriers. FAS trade data are collected and published online by the US Government. FAO TradeStat Detailed Trade Data [18] were used to obtain information on all imports into Central America (i.e. not just from the USA), although direct comparison with the FAS data (and with tariff changes) is not possible.

Information on tariff and non-tariff barriers, and changes in trade policies, was primarily sourced from USDA FAS Attaché Reports. These reports are prepared by in-country FAS officials, and are designed to assist US exporters in their assessment of overseas markets. Additional sources of information were the National Trade Estimate of the United States Trade Representative, the World Trade Organization's Trade Policy Review, trade policy analyses conducted by the USDA's Economic Research Service, the UNCTADs TRAINS database and academic articles.

### Analysis

Changes in tariff and non-tariff barriers for each country were documented, and were compared with time-series graphs of the import, production and availability data to identify the outcome of changes in trade policy in relation to food imports and food availability. Findings are presented for the five core food categories imported from the United States – staple grains and animal feed, meat, dairy, fruits and vegetables, and snacks – for the liberalizing period: 1990–2006.

## Results

### Overall changes in food imports, production and availability

**Figure 1:**
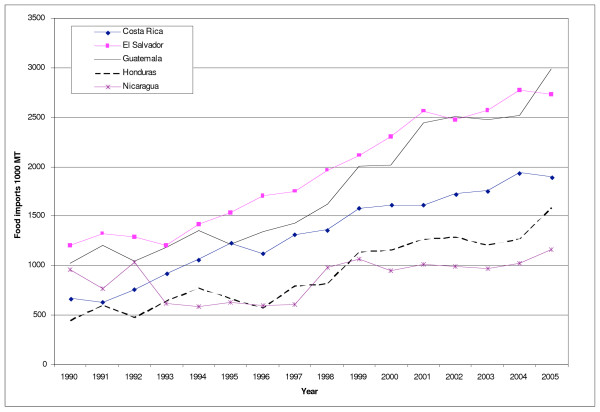
**Total food imports into the Central American countries, 1990–2005**. *Note*: "Food" includes animal meat (bovine, swine, sheep, poultry); fish; animal products (e.g. dairy products, eggs); vegetables; fruit; cereal grains; flours; raw nuts & seeds; fats & oils (some appear to be for industrial use, but are not split out for this spreadsheet); processed meats; sugar; cocoa beans & derivatives; cereal foods (processed); preserved foods (esp. vegetables); food preparations; non-alcoholic beverages. It excludes: live animals; inedible animal products (e.g. hair); plants, cut flowers etc; coffee, tea, spices; seeds definitely for planting etc; gums & saps; vegetable material (inedible); vegetable waxes & residues; alcohol & alcoholic drinks. Source [15].

Average tariffs in Central America declined from 45% in 1985 to around 6% in 2000. In line with this, total food imports into the Central American countries more than doubled between 1990/92–2003/05 from 4.5 to 9.6 million tonnes [19]. Honduras and Costa Rica registered the highest rates of growth, followed by Guatemala and El Salvador (Figure 1, Table 1). Food imports into Central America from the United States alone almost tripled since 1990 [17].

**Table 1:** Food imports, production and availability for consumption in the Central American countries, 1990/92–2003/05*, million metric tonnes

	**Average 1990/02**	**Average 2003/05**	**% increase 1990/92–2003/05**
**IMPORTS**	Million metric tonnes		
Costa Rica	0.7	1.9	173
El Salvador	1.3	2.7	112
Guatemala	1.1	2.7	144
Honduras	0.5	1.4	167
Nicaragua	0.9	1.1	15
Central America	4.5	9.6	115
**PRODUCTION**			
Costa Rica	7.4	11.0	49
El Salvador	6.0	7.0	16
Guatemala	15.2	24.4	61
Honduras	6.3	11.6	86
			
Nicaragua	3.9	6.6	67
Central America	38.8	60.6	56
**AVAILABILITY FOR CONSUMPTION**			
Costa Rica	3.4	4.8	40
El Salvador	4.0	5.0	24
Guatemala	6.1	8.2	35
Honduras	3.6	6.4	78
Nicaragua	2.6	3.5	37
Central America	19.6	27.9	42

Source: [19] * three-year average

Note: Changes in imports and production do not directly relate to changes in availability because of increases in food export and consumption by animals.

Between 1990 and 2005, the increase in the amount of food imported was relatively greater than the increase in production, indicating that imports became a more important source of foods consumed in the region (Table 1). Food available for consumption increased by less than the combined increase of production and imports, reflecting the fact that a greater proportion of the food supply is exported (90% increase between 1990 and 2005) or used as animal feed (75% increase). While these trends reflect the overall situation in Central America, there is a great deal of variation between the different food groups and countries, which are discussed below in relation to changing trade policies in the region.

### Staple grains

The United States is the leading source of imports of the three major grains, corn, rice, and wheat, into Central America. By volume, these grains comprise over 80% of all food imported from the United States [17], and imports have grown significantly since 1990, particularly of rice (Table 2).

**Table 2:** Imports of the three major grains into Central America from the United States, 1990/91 and 2005/06

	1990/91*, MT **(% of total)**	2005/06*, MT **(% of total)**	**% change** **1990–2006**
Yellow corn	562,071 (43%)	2,152,995 (51%)	283
White corn	0	204,733 (5%)	NA
Wheat	694,627(53%)	1,175,954 (28%)	69
Rice	64,623 (5%)	664,123 (16%)	928

Total	1,321,321	4,197,806	318

* 2-year averages because of zeros in data for 1989 and 2004; percentages do not add exactly due to rounding.

NA: Not Applicable

Source: [17]

**Figure 2:**
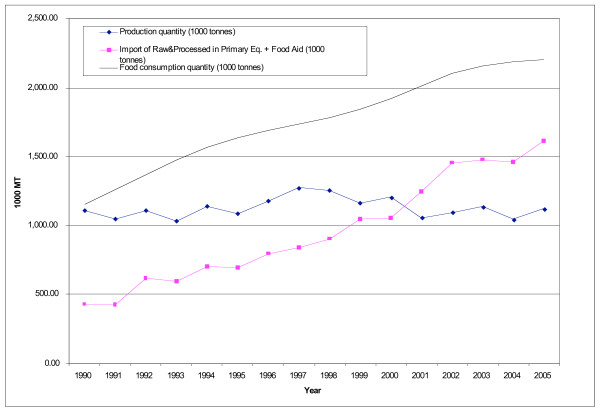
**Production, imports and consumption of rice in Central America, 1990–2005**. Source [15].

As rice imports have increased, domestic production has declined. However, the rise in imports has been greater than the decline of production, resulting in a greater overall level of supply, with rice availability increasing in all countries (Figure 2). In 1990, 39% of rice available for consumption in Central America was imported; the figure now stands at 69%. Over 90% of these imports are of rough rice (which needs to be milled before consumption).

Since rice is an important crop for domestic producers, it has historically been subject to high levels of protection and high tariffs remain in place (30–60%). Nevertheless trade policies for rough rice have been liberalized through alternate means: the removal of import licensing systems, the elimination of price banding mechanisms, the introduction of tariff-rate quotas, and the relaxation of phytosanitary requirements.

These new trade policies have had a clear impact on imports. In Honduras, for example, the replacement of the system of import licensing and administrative permits by a quota system in 1994 and relaxation of phytosanitary restrictions in 1997 were followed by a steady increase of rice imports [20-22]. In 1999, the government lowered the import tariff to 1%, further stimulating imports. In contrast, Nicaragua has had the smallest increase of rice imports in the region. Again, this reflects trade policies. In 1992, the government implemented a price band mechanism for rice, which directly restricted rice imports from the US and since then policies have remained restrictive. Notably, as a means of protecting the local rice milling industry – and in response to extensive lobbying by this industry – the market for milled rice has hardly been liberalized at all and imports have remained extremely low.

Trade policies have, then, facilitated greater availability of rice in the region, but with variation between countries due to policy differences. The situation for corn is a little more complex because there are two types of corn: yellow (animal feed) and white (human consumption). While corn imports into Central America have increased, this is overwhelmingly the result of increasing imports of yellow corn for animal feed (discussed in the next section) (Table 2). Imports of staple grain used for human consumption, white corn, remain limited due to high import barriers in place designed to protect domestic producers. These barriers continue under CAFTA implementation.

### Meat and animal feed

**Figure 3:**
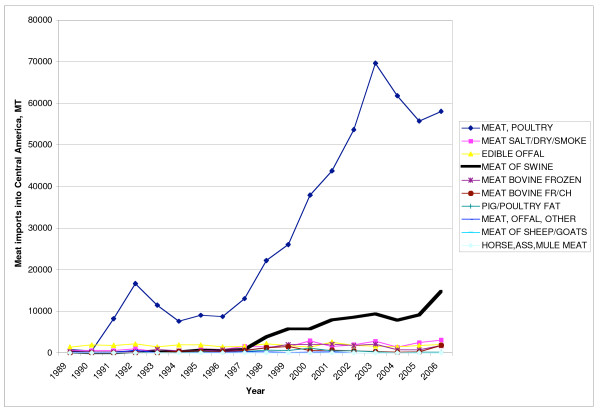
**Meat imports from the United States into Central America, 1989–2006**. Source [16].

The United States is the leading exporter of meat into Central America, and since 1990, meat exports have grown significantly (Figure 3). This largely reflects increasing exports of poultry and pork: poultry imports into Central America increased from 22% to 71% of total meat imports between 1990 and 2006, and pork imports from 6–18% (previously, imports were dominated by offal and preserved meat) (Figure 3). The steep increase of poultry imports is largely due to frozen poultry cuts, which now form 30% of all meat imports from the United States. Eighty-eight percent of these cuts are frozen chicken leg quarters, a by-product of chicken breast production in the United States [23].

Guatemala receives 90% of all poultry imported from the United States [17], and 58% of all chicken imports into the region [19]. In 2005, imported poultry from the United States represented approximately 30% of local consumption in Guatemala [24].

**Figure 4:**
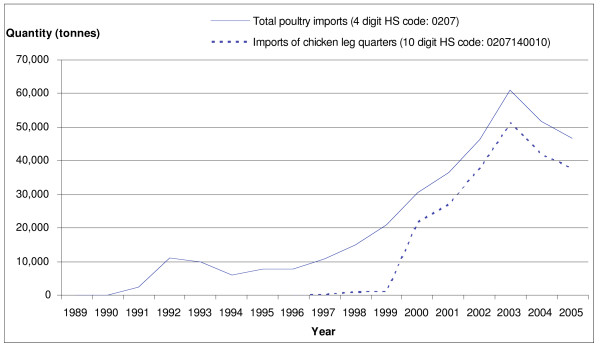
**Imports of chicken meat from the US into Guatemala, 1990–2005**. Source [16].

Imports of frozen chicken leg quarters into Guatemala grew particularly fast after 1997, a change that reflects the liberalization of trade policy, which progressed after the signing of the Peace Accords in 1996 [25]. Up until to 1997 (from at least 1995), there was a 3600 MT/year quota with a 20% in-quota tariff and 50% out-of-quota tariff, which created a strong disincentive to exporters [26]. However, in October 1996, the government announced a new poultry import policy that doubled the annual TRQ, and reduced the in-quota tariff to 15% [27]. Imports started to rise immediately (Figure 4). Reinforcing this policy, the TRQ was increased to 39,452 MT in 2005 with an in-quota applied tariff of 5%. According to analysis by the USDA "This greatly stimulated U.S. exports, and by 2005 poultry exports reached the highest value ever reported ($ 44.8 million)" [28]. As a result of the policy changes, "the growth in consumption is likely to have been picked up by US imports, leaving insignificant production growth" [29].

**Figure 5:**
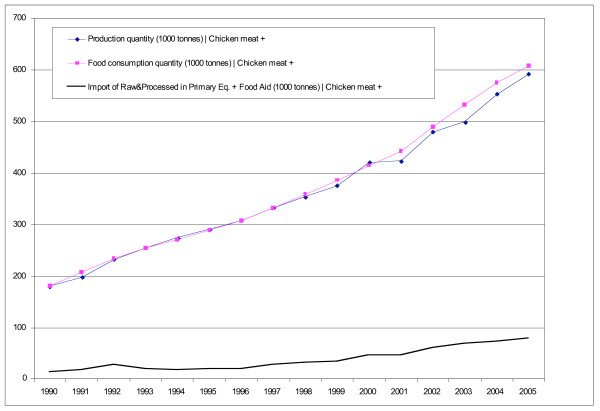
Production, imports, and availability for consumption of chicken meat into Central America, 1990–2005*. Source [15]. *The graph includes all chicken imports into the region, but the change since 1997 reflects imports from the United States.

Rising chicken imports into Guatemala have had a discernible impact on total chicken availability in the region (Figure 5). Reflecting much more restrictive import policies, imports into other countries have increased by a smaller amount. However, the limited import liberalization that did occur in the other Central American countries also boosted imports. For example, for most of the 1990s, Honduras implemented a 100% tariff on poultry meat and phytosanitary requirements restricted imports. In 1999, Honduras' tariff binding for poultry meat declined to 50%, and the country loosened its zoosanitary import requirements for poultry in an effort to comply with its WTO commitments [30,31]. Subsequently, poultry imports have increased by 20% per year and Honduras has emerged as the second largest chicken importer in the region [32].

Trade liberalization policies in Central America have clearly had an impact on chicken availability. However, the vast majority of increasing availability has been a result of increased domestic production (Figure 5). Yet this, too, partly reflects the impact of trade liberalization, since trade policies have stimulated the import of one of the major inputs into chicken production: yellow corn.

Imports of yellow corn into Central America from the United States increased by 283% between 1990 and 2006. During the same time period, most countries implemented limited but consistent measures to open up their market for yellow corn. In 1997, Guatemala, the leading corn importer in the region, opened up the TRQ for yellow corn imports, at a 5% in-tariff quota and a 55% out-of-quota tariff [33]. The quota was subsequently increased, reaching 100,000 MT by 2000 [34] and 501,820 MT in 2001 (5% in-tariff quota and out-of-quota tariff of 35%) [35].

**Figure 6:**
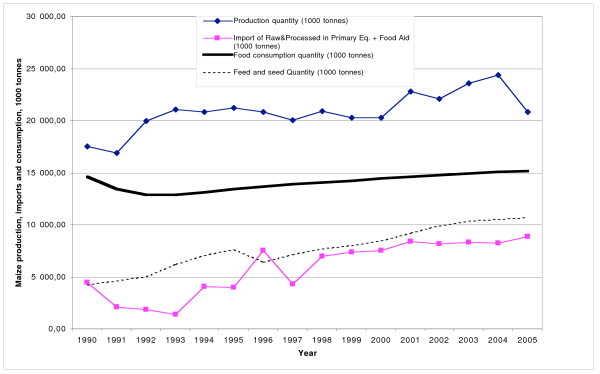
**Production, imports, consumption of corn (yellow and white) in Central America, 1990–2005**. Source [15].

The result has been increased availability of yellow corn for animal feed in the region (Figure 6). The increase cannot be explained by rising domestic production, since this is almost exclusively of white corn for human consumption. It is worth pointing out that the main user of yellow corn, the poultry sector, lobbied strongly for declines on import barriers for yellow corn. The reduction in the tariff on yellow corn implemented in El Salvador in 1995 was, for example, "mostly a result of pressure brought to bear on the government by poultry producers" [36].

### Dairy

In contrast to other commodities, the United States is not the leading dairy exporter into Central America: Europe and Australasia are important exporters, and there is considerable intra-regional trade. But between 1990/91 and 2004/06, imports of dairy products from the United States into Central America increased by 949%, and the United States became the leading exporter of two products: processed cheese and whey. Between 1990/91 and 2004/06, imports of processed cheese – such as cheese slices, sold in supermarkets and used by fast food outlets [37] – rose 3215% to comprise 37% of all cheese imports from the US [17]. It is notable that the two leading importers of processed cheese, Guatemala and Honduras, had significantly lower tariffs than the other countries: less than 20% compared with 35–66% in 2003 [38]. That these relatively high tariffs have clearly not been completely prohibitive, is likely to be because processed cheeses are predominantly sold to the fast food industry, or wealthier consumers able to afford higher prices in supermarkets.

The second product in which the United States dominates is whey – the liquid byproduct of cheese production – which formed 24.4% of all dairy product imports in 2004/06, an increase of 719% since 1989/91. The United States is the leading producer and exporter of whey in the world [39]. Whey and its derivatives are used in animal feed, pet foods, and as an ingredient in many processed foods [40]. The increase in imports is unlikely to have been directly affected by changing trade policies, since tariffs on whey in Central America have been consistently low; in 2003, tariffs were 0–1% for all countries [38]. Rather, increased imports reflect increased demand from the growing food processing industry in the region and extensive marketing by US whey exporters, with imports responding in the absence of trade barriers.

### Fresh and processed fruits and vegetables (including potatoes)

**Figure 7:**
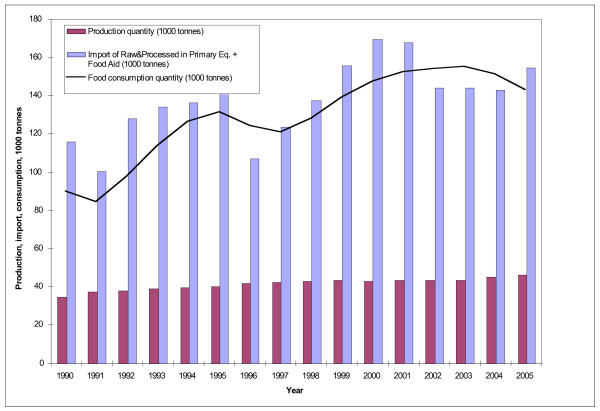
Imports of apples and grapes into Central America, 1990–2005*. Source [15]. * This graph shows imports into Central America from all countries, but imports are overwhelming dominated by the United States and Chile.

Imports of fresh fruits and processed fruits and vegetables from all countries into Central America have risen significantly since 1990, while imports of fresh vegetables have declined. Fresh fruit imports are largely (77%) of apples and grapes, of which the United States, alongside Chile, is the leading source of imports [18]. Although apples and grapes make up just 5% of total fruit consumption in the region, that they are consumed at all is a direct result of imports, since domestic production is low (Figure 7). In turn, increased imports have been associated with reduced trade barriers. Imports of apples into the regions' largest importing country, Guatemala, began to rise steeply in 1996, coinciding directly with the liberalization of the import market for apples through implementation of a TRQ and reduced in-quota tariff (12%). The new policy also eliminated the import licensing requirement for apples and allowed apple imports all year round [41]. Imports in 1996 filled the set quota; subsequent increases in imports reflect the higher TRQ of 10,000–15,000 MT set the following year [42].

**Figure 8:**
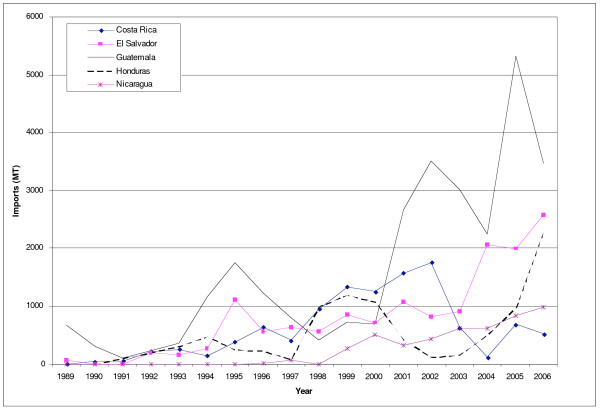
**Imports of french fries (frozen) into the Central American countries from the United States**. Source [16].

With regard to processed products, the most significant trend is the rise of imports of French fries, particularly post-2000 (Figure 8). French fries formed 23% of all imports of fruits and vegetables in 2004/06. The amount imported varies between countries: Guatemala led with 35% in 2006, compared to Costa Rica at 5%. The United States and Canada are the leading exporters of frozen potatoes to the region [18].

There are no data on availability of French fries in Central America, but if information from Costa Rica is illustrative, it is likely that all frozen French fries are imported, since domestic producers do not grow the specific type of potato required by the industry [43]. Thus imports are 100% responsible for availability. Sales of frozen French fries are largely to fast food outlets, restaurants and hotels. In Costa Rica, 75% of all frozen French fries enter this market, with the remaining 25% being sold by supermarkets [43]. In Guatemala, sales from supermarkets are apparently negligible, so it is likely that all imports are sold by the food service industry [37,44].

Tariffs on frozen French fries are not particularly high for four of the countries -15% – but it is notable that the country with the lowest amount of imports, Costa Rica, has a tariff of 41%. While imports into Costa Rica rose during the 1990s, fuelled by demand from fast food restaurants and the tourism industry, in the 2000s, imports from Canada grew rapidly to the detriment of other importers (Table 3). This was the direct result of policies arising from the Canadian-Costa Rica trade agreement, implemented in 2003. In the agreement, Costa Rica implemented a TRQ with a zero in-quota tariff for imports of Canadian French fries, with the 41% out-of-quota tariff phased out over eight years.

**Table 3:** Export Volume of Frozen French Fries to Costa Rica (2001–2005) (Metric Tons)

**Country**	**2001**	**2002**	**2003**	**2004**	**2005**
Canada	1,798	1,365	4,612	7,903	6,762

United States	2,156	2,039	456	324	866

Belgium	465	680	448	21	24

Netherlands	2.024	2,536	1,965	268	22

Others	28	211	60	0	159

Total	6,470	6,831	7,540	8,516	7,833

HS Code: 2041000

Source: [43]

No information could be obtained about trade policies specific to frozen French fries for the other countries. It is likely that increased imports stems from increased demand from the spread of the fast food industry in the region and the lack of a punitive tariff [45].

### Snacks

**Figure 9:**
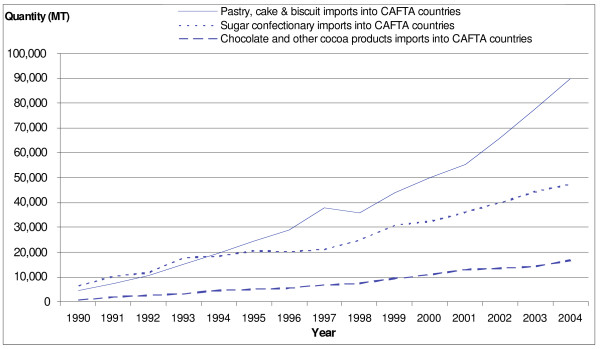
**Pastry, biscuit and confectionary imports into Central America, 1990–2004**. Source [14].

Snacks are defined by the USDA FAS data system as chocolate confectionary, sugar confectionary, chewing gum, cookies and pastries (sweet snacks) and popcorn, potato chips and other chips (savoury snacks). Imports of all snacks into Central America – as well as intra-regional trade – increased during the 1990s (Figure 9).

**Figure 10:**
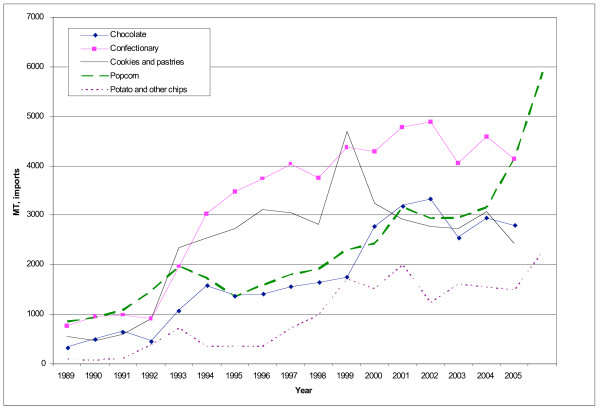
**Snack imports from the United States into Central America, 1989–2006**. Source [14].

Specifically, imports of chocolate, candy, cookies and pastries and popcorn from the United States into Central America grew in the early 1990s, and of potato and other chips in the late 1990s (Figure 10). As of 2006, the largest snack categories imported by weight were confectionary (chewing gum, sugar-based candy and chocolate) and popcorn.

There are no data on total availability of snacks in the countries, but expenditure data in two of the largest importing countries, Costa Rica and Guatemala, suggests that consumption is rising. In these two countries, sales of chips, popcorn, chocolate, confectionary and cookies all show a markedly increasing trend [37,44].

Tariffs on snacks into Central America are not notably high – all are under 20% with the exception of potato chips into Costa Rica, which faced a 41% tariff in 2003 [38]. Specific trade policy changes affecting snacks could not be identified from the available literature but trade barriers were reduced across the board in many Central American countries during the 1990s [46]. In addition, the growth of large supermarkets in the region – itself encouraged through the liberalization of investment policies – is likely to have increased the incentives for manufacturers to export into the region, particularly for commodities with low trade barriers [47-49]. Many of these supermarkets have established relationships with American processed food suppliers, and because of their size, capital base, economies of scale in storage and distribution and technological advancements in supply logistics, are able to make available a far wider range of snack foods relative to small stores [4].

It is also noteworthy that during the 1990s, the growth in processed food sales by US affiliates in Guatemala and Costa Rica significantly outstripped growth in sales of US exports [50]. Indeed, much of the market for chips in Guatemala is dominated by U.S. companies which have invested in the region. In 2005, PepsiCo had a 60% share of the market for sweet and savoury snacks [44]. US companies (Kraft, Mars, Hershey) also dominate the market for chocolate confectionary in both Costa Rica and Guatemala [37,44] This suggests that much of the market for snack foods from the United States is the result of foreign direct investment (FDI) into Central America by the food industry, rather than direct exports. American companies do, however, face significant domestic competition from leading snack food companies like Diana in El Salvador and Señorial in Guatemala. In cookies, for example, local companies have a greater market share than the U.S.-based Nabisco [44].

## Discussion

### Relationship between trade policy and food availability

Trade policy in Central American in the 1990s–2000s affected food availability through three key avenues. Firstly, the lowering of trade barriers is directly associated with increased imports which then, in most cases, leads to greater availability. Importers responded very quickly to changes in trade policy – for example, the sharp rise in imports of chicken cuts with reductions in tariffs, or the response of Canadian French fry manufacturers to the Costa Rican Free Trade Agreement – and are also able to take advantage of import opportunities in commodities with low barriers as market opportunities arise (e.g. whey imports with increased domestic food processing). These increased imports then, in most cases, are associated with increased availability of the food product. This is the case for both foods produced in large or small amounts in the importing region. For example, liberalization of trade policies in Central America contributed to the increased availability of rice, animal feed and fresh apples. Secondly, in the case of meat, trade liberalization affected food availability through its effects on domestic production. Lower barriers for yellow corn imports stimulated domestic chicken production, and may also have had implications for local corn farmers, given that US corn production is subsidized [51]. Thirdly, reductions in barriers to investment appear to be critical in expansion of processed food markets. Rising FDI has been a major driver of changes in availability of highly-processed foods and their ingredients (processed cheese, whey, French fries, snacks), so the relationship between availability and specific changes in tariff and nontariff barriers is less clear than that for agricultural commodities. This suggests the need for further work investigating changes in FDI in conjunction with trade liberalization.

Another key attribute of the impact of trade policy change on food availability highlighted by the analysis in this paper is that this is a two way relationship. While the food industry responds (often rapidly) to policy changes, it also shapes policy. In Central America, lobbying by producers and manufacturers affected the specifics of trade policy liberalization in relation to food. For example, lobbying by rice millers for reductions to import barriers for rough rather than milled rice, or chicken producers in relation to yellow corn.

This descriptive analysis has shown that the foods most affected by trade liberalization are those traditionally subject to high levels of protection. As the World Bank [10,15] has observed, these are both high value foods – such as poultry meat (both imports and domestic production), dairy, temperate fruits (apples and grapes), French fries and processed snack foods – and culturally significant foods, such as corn and rice. Many of these foods are also associated with the nutrition transition. As availability of animal products and processed foods has increased, this has been reflected in nutrition surveys indicating rising consumption of such 'transitional' foods [11,52,34]. Thus, the analysis suggests that food availability change associated with trade liberalization, in conjunction with social and demographic changes, has helped to facilitate dietary change in Central American countries towards increased consumption of meat, dairy products, processed foods and temperate (imported) fruits. It is also highly probably that there have also been decreases in the purchase price of these foods due to increased competition and economies of scale for producers and importers. Such dietary patterns have been associated with the nutrition transition and the growing burden of obesity and non-communicable disease reported in the region [53,54]. As such, addressing upstream drivers such as trade policy could form an essential part of strategies to improve population nutrition. Understanding the pathways through which trade policy has facilitated changes in food availability can help policy makers identify points of impact for potential interventions.

## Methods

This study has utilized a descriptive methodology for analyzing the relationship between trade policy and food availability. The strength of this method is the analysis of the relationship over time, using descriptive time series analysis of a variety of complimentary data sources, to identify responses to policy change. The weakness of the method is the inability to infer causality due to the descriptive nature of the analysis, or to estimate the relative importance of trade liberalization policies in driving change relative to other supply side drivers, such as technology, or demand side drivers, such as rising incomes. However, given the complexity of the interaction, the study was able to provide an initial level of evidence for the effect of trade policy on basic indicators of change in the food environment, and also to develop understanding of the pathways through which this impact occurs. Through this, possible policy levers can be identified that can be utilized in creating healthy trade policy.

Finally, while there are arguments for and against trade liberalization, it is essential to consider differential effects on the poor. Factors affecting income and distribution are important in determining diet and health, and these factors are likely to be more significant for the poor in the process of uneven dietary development [4,5].

## Conclusion

This analysis suggests that trade liberalization is one factor facilitating the nutrition transition, and indicates some of the processes and pathways through which this can occur. In Central America, it appears to have directly influenced the availability and price of meat and processed foods, many of which are energy-dense and high in fats, sugars and salt. Consumption of foods high in fat, salt and sugar, as well as consumption of animal products, are associated with increased rates of obesity and diet-related chronic diseases – in particular, cardiovascular disease, cancer and diabetes [1]. Trade liberalization therefore has direct implications for these health concerns. Indeed, evidence from Latin America already shows that the shift from traditional diets largely comprised of plant foods towards diets high in animal products and processed foods is associated with obesity and the shift of the burden of disease towards cardiovascular disease and cancer [55-57]. Compounding this health transition, in many developing countries under and over-nutrition co-exist (sometimes even within the same household) [58]. The costs of such chronic diseases have been well documented, not only for the healthcare system but also for individuals, families and communities, as has the need for intervention at a macro level [59].

As these foods become more readily available and increasingly affordable – and social trends continue to favour convenience and 'transitional' foods – the burden of diet-related chronic disease will continue to grow. An analysis of the implications of full implementation of CAFTA-DR in Central America has shown that the trade agreement is likely to further the nutrition transition by exacerbating the trends in food availability observed in this study of previous trade liberalization measures [60]. In particular, iberalization of trade, investment and communications associated with CAFTA-DR is likely to increase availability and lower relative prices of meat and processed foods, and through this may continue to facilitate the rising prevalence of obesity and chronic disease.

Efforts to mitigate the negative impacts of trade policy on diets – including engagement and negotiation with trade policy makers – must begin with an understanding of how the processes of globalization have facilitated the nutrition transition. This paper provides evidence for the pathways and processes through which trade liberalization affects food availability and price, and thus equips public health advocates to effect policy change.

## Competing interests

The authors declare that they have no competing interests.

## Authors' contributions

AMT participated in the study design, conducted the data analysis and drafted the manuscript. CH conceived of the study, and participated in its design and coordination and helped to draft the manuscript. Both authors read and approved the final manuscript.

## Authors' informations

At the time of the study, CH was a research fellow and AMT an intern at the International Food Policy Research Institute. AMT is presently at the Menzies Centre for Health Policy, University of Sydney School of Public Health. CH is a Research Fellow at the School of Public Health, University of Sao Paolo, Brazil.

## References

[B1] Popkin B (2006). Global nutrition dynamics: the world is shifting rapidly toward a diet linked with noncommunicable diseases. Am J Clin Nutr.

[B2] Popkin BM (1998). The nutrition transition and its health implications in lower income countries. Public Health Nutr.

[B3] Nugent R (2004). Food and agriculture policy: issues related to prevention of noncommunicable diseases. Food Nutr Bull.

[B4] Hawkes C (2006). Uneven dietary development: linking the policies and processes of globalization with the nutrition transition, obesity and diet-related chronic diseases. Global Health.

[B5] Schmidhuber J, Shetty P (2005). Nutrition transition, obesity and noncommunicable diseases: drivers, outlook and concerns. SCN News.

[B6] Popkin BM (2006). Technology, transport, globalization and the nutrition transition. Food Policy.

[B7] Thow AM (2009). Trade liberalisation and the nutrition transition: mapping the pathways for public health nutritionists. Public Health Nutr.

[B8] Hawkes C, Chopra M, Friel S, Lang T, Thow AM (2007). Globalization, food and nutrition transitions.

[B9] Blouin C, Chopra M, Hoeven R van der (2009). Trade and health 3: trade and the social determinants of health. Lancet.

[B10] Jaramillo CF, Lederman D (2005). DR-CAFTA: challenges and opportunities for Central America.

[B11] Stein AD, Gregory CO, Hoddinott J, Martorell R, Ramakrishnan U, Ramirez-Zea M (2005). Physical activity level, dietary habits, and alcohol and tobacco use among young Guatemalan adults. Food Nutr Bull.

[B12] Bermudez OI, Tucker KL (2003). Trends in dietary patterns of Latin American populations. Cad Saude Publica.

[B13] Torun B, Stein AD, Schroeder D, Grajeda R, Conlisk A, Rodriguez M, Mendez H, Martorell R (2002). Rural-to-urban migration and cardiovascular disease risk factors in young Guatemalan adults. Int J Epidemiol.

[B14] FAO (2007). FAOSTAT Core Production Data.

[B15] FAO (2007). Food Balance Sheets (archive).

[B16] FAO Supply Utilization Accounts and Food Balance Sheets in the context of a national statistical system.

[B17] US Department of Agriculture (2007). Foreign Trade Statistics.

[B18] FAO (2007). FAOSTAT Detailed Trade Matrix.

[B19] FAO (2007). FAOSTAT SUA Data.

[B20] USDA FAS (United States Department of Agriculture Foreign Agricultural Service) (1995). Rice import policy update: AGR Number: HO5009.

[B21] USDA FAS (United States Department of Agriculture Foreign Agricultural Service) (1995). Rice export possibilities are enhanced.

[B22] USDA FAS (United States Department of Agriculture Foreign Agricultural Service) (1997). Rice update.

[B23] Dyck J, Nelson K (2000). World Meat Trade Shaped by Regional Preferences & Reduced Barriers. Ag Outlook (USDA).

[B24] Tay K, Huete SM (2005). Guatemala: poultry and products, production and consumption.

[B25] WTO (2002). Guatemala Trade Policy Review.

[B26] Aguilar V, Drennan RT (1996). Poultry, Annual Report.

[B27] Aguilar V, Gonzalez A, Heinen SE (1997). Agricultural situation: Guatemala.

[B28] Tay K, Huete SM (2006). Guatemala poultry and products, production and consumption 2006.

[B29] USDA FAS (United States Department of Agriculture Foreign Agricultural Service) (2001). Guatemala Poultry and Products Tariff Reduction 2001.

[B30] Gonzalez O, Heinen SE (1998). Poultry annual report.

[B31] USDA FAS (United States Department of Agriculture Foreign Agricultural Service) (1999). Honduras Poultry Annual Report 1999.

[B32] Ovalle D, Coolidge F (2006). Honduras: poultry and products annual 2001.

[B33] USDA FAS (United States Department of Agriculture Foreign Agricultural Service) (1996). Yellow corn tariff lowered.

[B34] Ovalle D, Coolidge F (2001). Guatemala food and agricultural import regulations and standards: tariff and quota changes in yellow corn, wheat and rice imports.

[B35] USDA FAS (United States Department of Agriculture Foreign Agricultural Service) (2001). Guatemala Poultry and Products Annual 2001: GAIN Report #GT1020.

[B36] Pettrie GA (1995). Grain and feed: corn tariff reduction and no return to price band.

[B37] Euromonitor (2007). Costa Rica – Packaged Foods Euromonitor.

[B38] CAFTA Agreement (2004). Annex 33 – Tariff Schedules.

[B39] Coggin B (1998). The Best Whey To Expand Exports. AgExporter.

[B40] USDA FAS (United States Department of Agriculture Foreign Agricultural Service) (1999). US Whey Exports.

[B41] USDA FAS (United States Department of Agriculture Foreign Agricultural Service) (1996). Fresh Deciduous Fruit.

[B42] USDA FAS (United States Department of Agriculture Foreign Agricultural Service) (1997). Fresh Deciduous Fruit.

[B43] USDA FAS (United States Department of Agriculture Foreign Agricultural Service) (2007). Costa Rica Market Development Reports: frozen foods report.

[B44] Euromonitor (2007). Guatemala – Packaged Foods Euromonitor.

[B45] Montel JE (1996). Frozen foods gain warm welcome in Guatemala and El Salvador. AgExporter.

[B46] Pettrie GA (1995). CA tariff reductions should boost US agricultural exports.

[B47] Herrera M, Orellana D, Suazo H (2001). Three Central American markets add up to opportunities for US food exporters. AgExporter.

[B48] Reardon T, Berdegué JA (2002). The rapid rise of supermarkets in Latin America: challenges and opportunities for development. Dev Policy Rev.

[B49] Orellana D, Huete SM (2004). Guatemala exporter guide, annual 2004.

[B50] Mattson JW, Koo WW (2002). US processed food exports and foreign direct investment in the Western hemisphere.

[B51] Morley S (2006). Trade liberalization under CAFTA: an analysis of the agreement with special reference to agriculture and smallholders in Central America DSGD Discussion Paper No33/MTID Discussion Paper No95.

[B52] Marini A, Gragnolati M (2003). Malnutrition and Poverty in Guatemala: World Bank Policy Research Working Paper 2967.

[B53] Martorell R, Kettel Khan L, Hughes ML, Grummer-Strawn LM (1998). Obesity in Latin American women and children. J Nutr.

[B54] Lanas F, Avezum A, Bautista LE, Diaz R, Luna M, Islam S, Yusuf S, INTERHEART Investigators in Latin America (2007). Risk factors for acute myocardial infarction in Latin America: the INTERHEART Latin American study. Circulation.

[B55] Albala C, Vio F, Kain J, Uauy R (2001). The nutrition transition in Latin America: the case of Chile. Nutr Rev.

[B56] Rivera JA, Barquera S, Campirano F, Campos I, Safdie M, Tovar V (2002). Epidemiological and nutritional transition in Mexico: rapid increase of non-communicable chronic diseases and obesity. Public Health Nutr.

[B57] Kain J, Vio F, Albala C (2003). Obesity trends and determinant forces in Latin America. Cad Saúde Pública.

[B58] Garrett JL, Ruel MT (2005). Stunted child-overweight mother pairs: Prevalence and association with economic development and urbanization. Food Nutr Bull.

[B59] WHO (World Health Organization) (2005). Preventing chronic disease: a vital investment.

[B60] Hawkes C, Thow AM (2008). Implications of the Central America-Dominican Republic-Free Trade Agreement for the nutrition transition in Central America. Rev Panam Salud Publica.

